# Three *Factor 11* Mutations Associated with Factor XI Deficiency in a Turkish Family

**DOI:** 10.4274/tjh.2017.0140

**Published:** 2018-03-06

**Authors:** Veysel Sabri Hançer, Zafer Gökgöz, Murat Büyükdoğan

**Affiliations:** 1 İstinye University Faculty of Medicine, Department of Medical Genetics, İstanbul, Turkey; 2 Medicana International Ankara Hospital, Clinic of Hematology, Ankara, Turkey

**Keywords:** Factor XI, Mutation, Family

## To the Editor,

Factor XI (FXI) is a homodimeric serine protease, which is produced in the liver and circulates in the plasma complexed with high-molecular-weight kininogen. FXI plays an important role in the amplification of the initial coagulation response via a positive feedback mechanism for the generation of additional thrombin [1,2,3,4]. Congenital FXI deficiency is characterized by decreased levels or activity of FXI in the plasma and may cause an inherited bleeding disorder. The FXI gene is located on 4q34-35 and consists of 15 exons. 

The index case was a 10 year-old-boy with bleeding diathesis (excessive bleeding after tooth extraction). His activated partial thromboplastin time (aPTT) was 84.3 s (normal range: 32-39 s), FXI activity was 0%, and he was diagnosed with FXI deficiency. His parents were related. The father had a mild bleeding tendency with prolonged aPTT (48.2 s). FXI activity was found to be 4%. The mother and the second child had no bleeding history with mildly decreased FXI activities (40% and 60%, respectively). We performed a mutational analysis for the whole family, including the patient’s grandparents. Genomic DNA was extracted from whole blood. All exons and approximately 25-bp exon-intron boundaries of the factor 11 (*F11*) gene were amplified using sets of designed primers. After polymerase chain reaction, the amplified fragments were sequenced.

**Figure 1:**
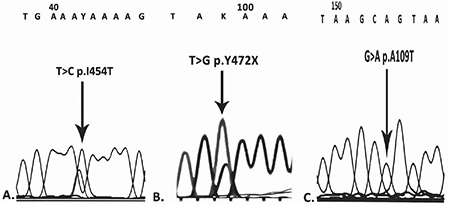
Electropherogram results.

**Figure 2:**
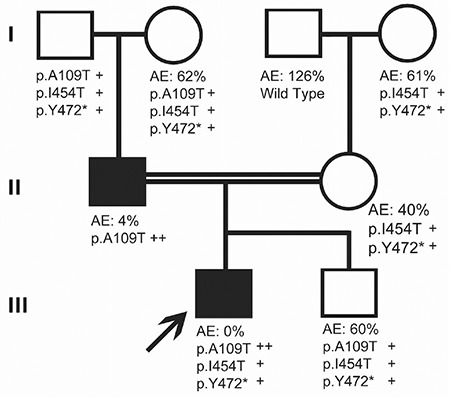
Pedigree of the family. *AE: Activity of the enzyme, +: heterozygous, ++: homozygous.*

The patient and his father had a p.Ala109Thr (ENST00000492972.6, p.A109T, c.325 G>A, rs768474112) homozygous mutation for *F11*; the patient also had novel heterozygous p.I454T and p.Y472* mutations (Figure 1). The presence of a homozygous p.A109T mutation in the father and the index patient caused severe FXI deficiency. The mother and the second child had heterozygous p.I454T and p.Y472* mutations. As shown in Figure 2, p.I454T and p.Y472* heterozygosity moderately decreases the activity of FXI. In this family, we found two novel mutations, p.I454T and p.Y472*, associated with a homozygous p.A109T mutation. p.I454T is probably damaging with a PolyPhen score of 0.9. This is the first case reported in the literature with homozygous p.A109T. Previously, Guella et al. [5] reported a heterozygous p.A109T mutation in an Italian family with FXI deficiency. They showed that exon-skipping had occurred due to a heterozygous p.A109T mutation and they explained that the unchanged enzyme activity was due to a non-sense mediated RNA decay mechanism. With this mechanism, due to p.A109T mutation, incorrectly spliced transcripts are not allowed to exit the nucleus to the cytoplasm. Our cases confirmed their results, such that a heterozygous p.A109T mutation did not affect enzyme activity; the enzyme activity of a person who has two heterozygous mutations (p.Y472* and p.I454T) is the same as that of someone who has three heterozygous mutations (p.A109T, p.Y472*, and p.I454T). However, when p.A109T was homozygous, like in our index patient and his father, the enzyme activity decreased by approximately 96% as shown in the pedigree. Another interesting point was the presence of a homozygous p.A109T mutation in the patient while his mother had no p.A109T mutation. This may be explained by a second-hit de novo mutation in the index case. Further expression studies evaluating the effects of these mutations will improve our understanding of the functional and structural features of the FXI enzyme.
